# Supplementary material for: Biomarker-Based Risk Assessment Strategy for Long COVID: Leveraging Spike Protein and Proinflammatory Mediators to Inform Broader Postinfection Sequelae
Source: Viruses. 2025 Sep 5;17(9):1215. doi: 10.3390/v17091215 (PMC12474022; doi:10.3390/v17091215)
Supplement: Supplementary file 1 [file viruses-17-01215-s001.zip › viruses-3800102-supplementary.pdf]

Supporting material

Figure S1. Raw data of temporal profiling of SARS-CoV-2 spikes in post-acute sequelae of COVID-19 (PASC) (n=12) and non-PASC patients (n=6).
Figure S2. Scree plot showing variabilities (within groups sum of squares) in clusters. Solid line is a guide to the eyes.
Table S1. Experimental data of relationships between cycle threshold C_t value and SARS-CoV-2 viral load concentration (log copy μL^{-1}) adopted from Girón Pérez et al. [1].
Table S2. Experimental data of relationships between SARS-CoV-2 viral load concentration (log copy μL^{-1}) and symptom numbers after COVID-19 infection adopted from Girón Pérez et al. [1].
Table S3. Fitted coefficients (mean±S.E.) of three-parameter Hill model describing symptom numbers of Long COVID corresponding to different viral loads
Table S4. Fitted coefficients (mean±S.E.) of mathematical model ($y=a+bx^2+ce^x+dx/\ln x$) describing months post diagnosis-dependent spike concentration (pg mL^{-1})
Table S5. Fitted coefficients (mean ± S.E.) of the three-parameter Hill model describing fraction of protein expression compared to LPS (1 $\mu g\ mL^{-1}$) in human lung macrophage treated with different spike protein concentrations

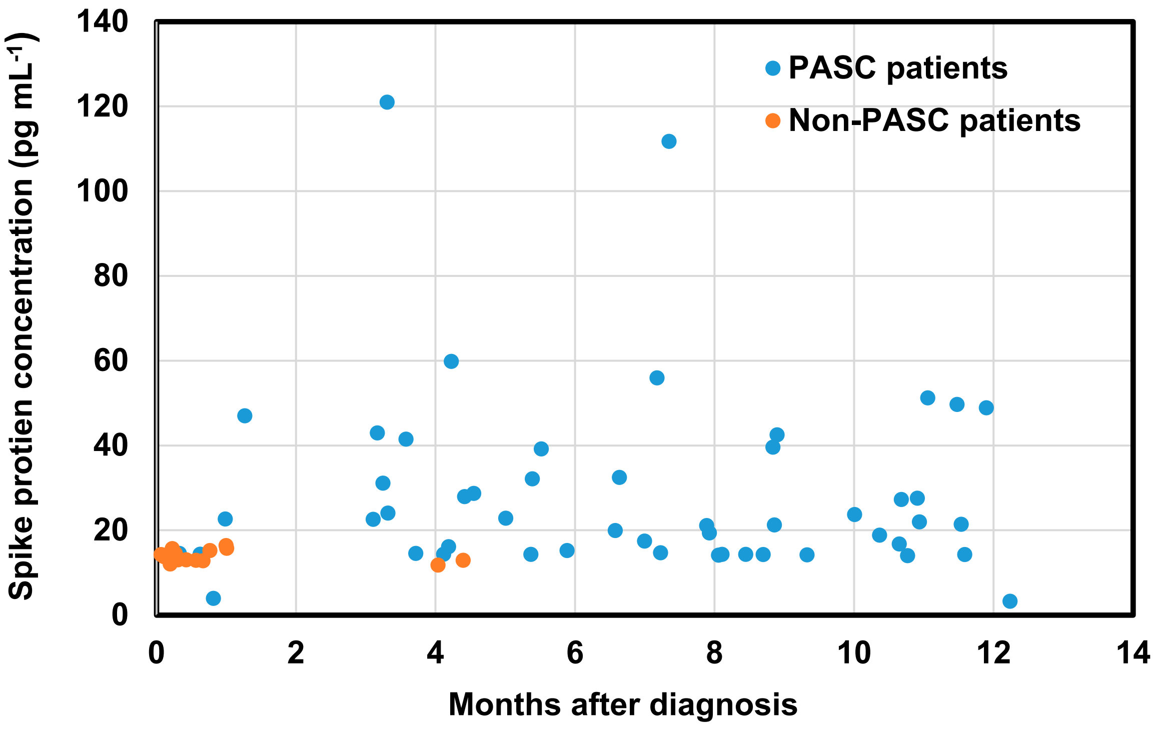

Figure S1. Raw data of temporal profiling of SARS-CoV-2 spikes in post-acute sequelae of COVID-19 (PASC) (*n*=12) and non-PASC patients (*n*=6).

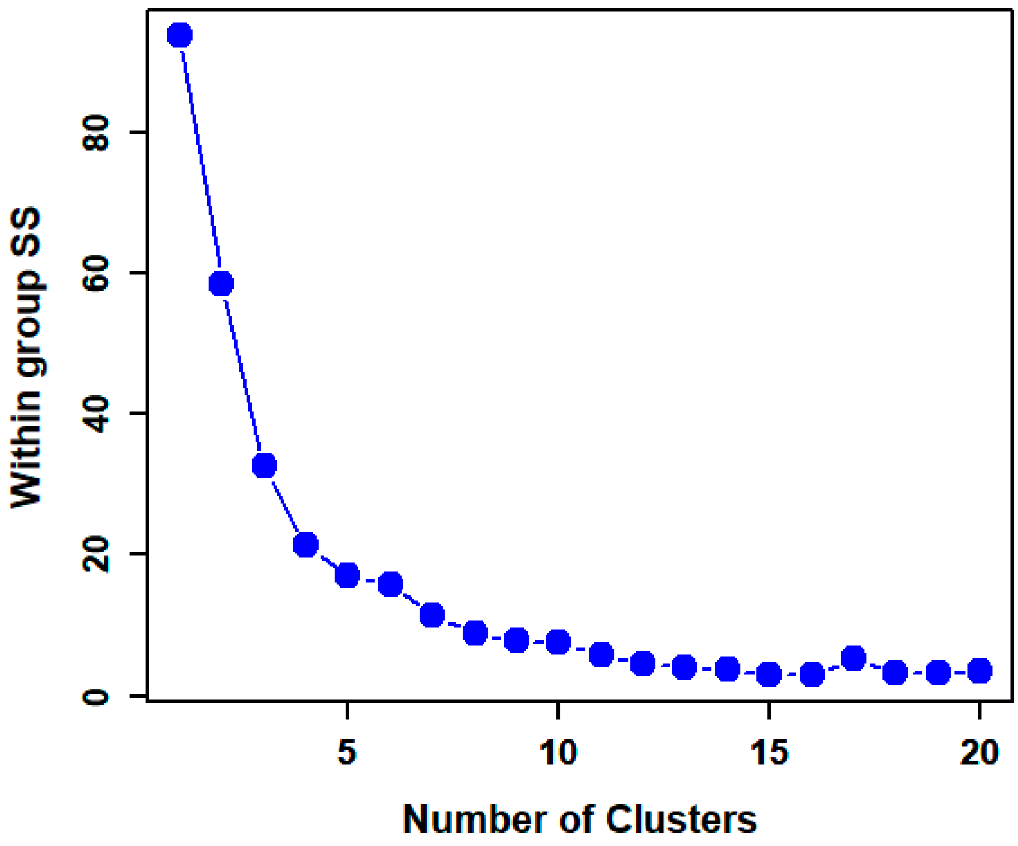

Figure S2. Scree plot showing variabilities (within groups sum of squares) in clusters. Solid line is a guide to the eyes.

Table S1. Experimental data of relationships between cycle threshold C_t value and SARS-CoV-2 viral load concentration (copy μL^{-1}) adopted from Girón Pérez et al. [1]

C_t	copy μL^{-1}
20<	<451,743
20	451,743
21	241,631
22	129,245
23	69,131
24	36,977
25	19,779
26	10,579
27	5659
28	3027
29	1619
30	866
31	463
32	248
33	133
34	71
35	38
36	20
37	11
38	6
39	3
40	2

Table S2. Experimental data of relationships between SARS-CoV-2 viral load concentration (log copy μL^{-1}) and symptom numbers after COVID-19 infection adopted from Girón Pérez et al. [1]

Log copy μL^{-1}	Symptom numbers after COVID-19 infection
0.001488	0
0.001498	2
0.003123	1
0.003123	0
0.003144	3
0.003144	4
0.003293	2
0.005937	7
0.006177	0
0.011668	3
0.011746	5
0.012468	7
0.023246	4
0.02340	6
0.046017	3
0.086982	4
0.164453	6
0.362295	6
0.624478	12
1.237735	11
1.237735	12
1.432527	13
2.342329	15
2.357947	16
2.453868	12
4.433700	13
4.583553	7
4.614126	10
8.735993	11
8.794285	13
9.213372	12

Table S2 (Continued)

9.274851	15
17.68181	18
18.2799	11
18.40192	13
34.39681	9
34.39681	8
34.62651	12
65.17044	10
128.5396	5
128.5396	7
130.2633	14
131.1338	15
136.4805	10
245.2826	12
260.4412	16
262.1823	18
490.5238	13
493.8043	17
497.1067	20
930.2577	15
981.2274	16
987.7921	17
1861.316	18
1937.321	12
1950.287	14
2057.191	15
3651.401	10
3700.458	16
3929.505	20
3929.505	18

Table S3. Fitted coefficients (mean ± S.E.) of three-parameter Hill model describing symptom numbers of Long COVID corresponding to different viral loads

Hill-based parameters	Estimate
E_{max}	19.83 ± 5.44
$ED50$ (log copyμL^{-1})	3.77 ± 1.27 (~1.8 × 10^{-6} μg mL^{-1} (assuming spike monomer MW = 180 kDa)) [2]
n	1.93 ± 0.62
r^2	0.67
p-value	***

***$p < 0.001$.

Abbreviations: E_{max} is the maximum symptom numbers, $ED50$ is the viral dose (Log copy μL^{-1}) corresponding to an effect equal to 50% E_{max}, and n is the fitted Hill coefficient.

Table S4. Fitted coefficients (mean ± S.E.) of mathematical model ($y=a+bx^2+ce^x+dx/\ln x$) describing months post diagnosis-dependent spike concentration (pg mL^{-1})

Model parameters	Estimate
a	987.38 ± 33.04 (**)
b	7.16 ± 0.24 (**)
c	-0.003 ± 8.55×10^{-5} (**)
d	-367.57 ± 12.55 (**)
r^2	0.99
p-value	***

**$p < 0.01$; ***$p < 0.001$.

Table S5. Fitted coefficients (mean ± S.E.) of the three-parameter Hill model describing fraction of protein expression compared to LPS at 1 μg mL^{-1} in human lung macrophage treated with different spike protein concentrations

	CXCL8	IL-1β	IL-6	TNF-α
E_{max}	0.72 ± 0.15	0.88 ± 0.09	0.16 ± 0.01	0.06 ± 0.00
$ED50$ (μg mL^{-1})	0.01 ± 0.01	0.46 ± 0.22	0.39 ± 0.16	0.56 ± 0.09
n	0.50 ± 0.52	2.00 ± 1.02	1.67 ± 0.57	1.61 ± 0.35
r^2	0.38	0.85	0.88	0.98
p-value	0.06	***	***	***

*** $p < 0.001$.

Reference

1. Girón Pérez, D.A.; Fonseca-Agüero, A.; Toledo-Ibarra, G.A.; Gomez-Valdivia, J.J.; Díaz-Resendiz, K.J.G.; Benitez-Trinidad, A.B.; Razura-Carmona, F.F.; Navidad-Murrieta, M.S.; Covantes-Rosales, C.E.; Giron-Pérez, M.I. Post-COVID-19 syndrome in outpatients and its association with viral load. *Int. J. Environ. Res. Public Health* 2022, *19*, 15145.
2. Huang, Y.; Yang, C.; Xu, X.F.; Xu, W.; Liu, S.W. Structural and functional properties of SARS-CoV-2 spike protein: Potential antivirus drug development for COVID-19. *Acta Pharmacol. Sin.* 2020, *41*, 1141–1149.
